# 5G Security Threat Assessment in Real Networks

**DOI:** 10.3390/s21165524

**Published:** 2021-08-17

**Authors:** Seongmin Park, Daeun Kim, Youngkwon Park, Hyungjin Cho, Dowon Kim, Sungmoon Kwon

**Affiliations:** Infrastructure Security Technology Team, Korea Internet & Security Agency, 9, Jinheung-gil, Naju-si 58324, Korea; smpark@kisa.or.kr (S.P.); whale53@kisa.or.kr (D.K.); youngk6874@kisa.or.kr (Y.P.); hjcho86@kisa.or.kr (H.C.); kimdw@kisa.or.kr (D.K.)

**Keywords:** 5G mobile communication, non-standalone, mobile network, RRC, NAS, GTP, SIP, 5G security testing

## Abstract

Advances in mobile communication networks from 2G to 5G have brought unprecedented traffic growth, and 5G mobile communication networks are expected to be used in a variety of industries based on innovative technologies, fast not only in terms of extremely low latency but massive access devices. Various types of services, such as enhanced mobile broadband (eMBB), massive machine type communication (mMTC), and ultra-reliable and low latency communication (uRLLC), represent an increase in the number of attacks on users’ personal information, confidential information, and privacy information. Therefore, security assessments are essential to verify and cope with these various attacks. In this research, we (1) looked at 5G mobile communication network backgrounds and problems to investigate existing vulnerabilities and (2) assessed the current situation through evaluation of 5G security threats in real-world mobile networks in service.

## 1. Introduction

In February 2017, international telecommunication union (ITU) released a report that established key requirements which is minimum requirements related to technical performance for IMT-2020 for 5G mobile communication technology [1]. This report requested 1 GHz of minimum bandwidth, 20 Gbps of maximum data transmission rate, and 1 ms of the shortest latency time for next-generation services. These are technical requirements to realize the key purposes of 5G: super-connection, ultra-fast, and ultra-low latency, and also the minimal requirements to realize various 5G services.

5G mobile communication is more innovatively advanced compared to 4G mobile communication in general including speed, using protocol, and network configurations. 5G wireless network is configured in soft defined network (SDN) with 20 Gbps speed, 20 times faster than existing long term evolution (LTE), while the 5G core network has been changed from a centralized type to a decentralized type to minimize traffic transmission delay.

Owing to such technical changes, ITU-R defined 5G services. It classified 5G services into enhanced mobile broadband (eMBB) where speed is a key element, massive machine type communication (mMTC) where bandwidth is a key element, and ultra-reliable and low latency communication (uRLLC) where latency time minimization is required, and configured the services to use 5G infrastructures in industrial environment throughout society [2].

5G technical standards are led by 3rd Generation Partnership Project (3GPP), and release 15 frozen in March 2019 defines the architectures of non-standalone (NSA) and standalone (SA) and covers LTE system migration. In addition, release 16 frozen in July 2020 covers 5G-based convergence industry support including 5G-vehicle to everything communication (V2X) and 5G internet of things (IoT), along with performance improvements in 5G system. In release 17, which will be frozen in March of 2022, standardization is underway with the aim of expanding 5G coverage, transmitting small data, and utilizing unlicensed bands. Additionally, as sales of 5G-related industries are on the rise, sales of 5G wireless network infrastructures are expected to reach USD 6.8 billion in 2021 according to reports published by the Gartner as shown in Table 1 [3].

In this context, the voices of users calling for a safe 5G service environment are growing, and many reviews are required before launching the services because of the possibility of inherent security threats in existing LTE communication network. Additionally, new 5G security threats need to be identified in advance due to technical changes differentiated from 4G, and service safety must be strengthened by developing security technology dedicated to 5G, if necessary.

In addition, the scale of the global 5G security market is expected to grow at 50% of the compound annual growth rate (CAGR) to about USD 4 billion in 2023 and about USD 7 billion in 2025. In particular, more than 95% of the 5G security market is occupied by the field of security solutions [4]. Recently, serious security threats are being reported in papers such as Hongil Kim et al. and Merlin Chlosta et al. [5,6]. However, most mobile carriers (5G service operator) are providing 5G services without applying techniques of reaction related to security threats.

The contribution of this paper is as follows and the main contribution is that we provide insight into what security challenges are valid in the real 5G NSA network and how they could be mitigated.

- We divided 5G NSA security threats into radio access network (RAN) and core network (CN) to create an attack tree and develop 15 test cases that can be applied to real networks.
- We verified the developed 15 test cases on the actual three mobile carriers’ networks and identifies eight valid vulnerabilities.
- Of these eight valid vulnerabilities, we proposed equipment PKG software patches or configuration changes for five and relevant countermeasures for the remaining three.

The composition of this paper is as follows. Section 2 describes the background of 5G NSA architecture and attachment procedure, while Section 3 describes related works on 5G NSA security. Section 4 describes security challenges along with the attack tree for 5G NSA network, and Section 5 presents the test cases of security threats for the security challenges in the Section 4. Section 6 describes implementation and environment for the test, and Section 7 describes vulnerability action in real networks along with the test results. Section 8 describes countermeasures and future works, and, finally, Section 9 concludes this paper.

## 2. Background

This section covers Section 2.1 5G NSA architecture and the Section 2.2 5G NSA terminal attachment procedure.

In terms of traditional network security, a mobile communication network is a very inaccessible infrastructure. Such inaccessibility can be discussed in three aspects, and the first of them is complicated network configuration. Mobile communication networks can be divided broadly into wireless network, core network, and interconnection network sections, and protocols and interfaces used in each section are different. This means that different security technologies must be applied and managed for each section. Additionally, there are so many sections that security monitoring points is complicated, and traffic analysis to find security threats is inevitably difficult [7].

The second is that the terminals use private internet protocol (IP) address, unlike general networks. Because the terminal IP address changes whenever the terminal accesses the mobile communication network, even if a security threat is found, it is very difficult to identify the attacker who caused that threat [8].

The last is the use of dedicated protocols. There is a limit to enhancing stability by utilizing existing security equipment that cannot interpret the dedicated protocols, because it uses protocols used only in mobile communication networks such as NG application protocol (NGAP), non-access stratum (NAS), GPRS tunneling protocol (GTP), diameter, and packet forwarding control protocol (PFCP) rather than general transmission control protocol (TCP)/IP. In particular, because the 5G mobile communication network uses different core network dedicated protocols in both NSA and SA configurations, the security requirements inevitably vary depending on the network configuration [9].

### 2.1. 5G NSA Arthitecture

5G NSA is a method where the core network is configured as LTE-based evolved packet core (EPC), and both evolved node B (eNB) and next generation node B (gNB) are used together for wireless networks. Given that it is difficult to quickly introduce 5G SA method from the perspective of mobile carriers who provided existing LTE services, soft landing is easy for 5G services in this architecture.

**Figure 1:**
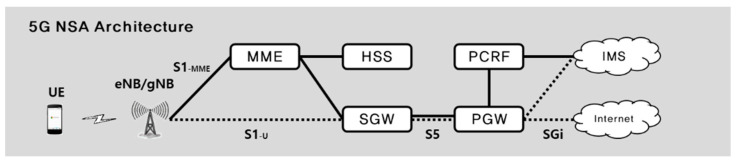
5G NSA architecture.

5G NSA communication section is divided into a wireless section and a wire section as shown in Figure 1. The wireless section represents RAN between User Equipment (UE) and base station, while the wire section represents CN between base station and service network. Here, there is a control plane (CP) between a terminal and RAN or CN, while there is a user plane (UP) between a terminal and IP multimedia subsystem (IMS) network for internet or voice services. In the CP, traffic using protocols such as radio resource control (RRC), NAS, and GTP-C is transmitted, while tunneling traffic using the GTP-RRC protocol in user data, such as a voice, is transmitted in the UP. Figure 1 shows 5G NSA architecture with a simplified configuration based on components required in this paper. The descriptions of each component in 5G NSA are as follows [10].

- **User equipment (UE)**: User terminals and includes smartphones, USB modems, computers with built-in mobile communication modules.
- **Evolved node B (eNB)**: Provides wireless interface to UE and is used for functions related to UE control in 5G.
- **Next generation node B (gNB)**: Provides wireless interface to UE and is used for data transmission.
- **Mobility management entity (MME)**: Manages authentication and connection status, and active status for UE.
- **Home subscriber server (HSS)**: A central database that manages key information and subscriber profile for authentication for each UE. When an UE accesses the network, it delivers relevant key information and subscriber profile for UE authentication to MME.
- **Serving gateway (S-GW)**: Routes and delivers user packets between the base-station and P-GWs, and plays a role of anchoring point when the UE performs handover between eNBs or gNBs.
- **Packet data network gateway (P-GW)**: Connects the UE to an external packet data network (PDN). It acts as a channel for delivery packets between the UE and the PDN, and performs functions such as charging according to data usage and allocating IP address to the UE.

Unlike NSA, new technical elements should be considered in accessing 5G SA network security. Wireless networks, called 5G NR, have changed the configuration form from existing radio unit (RU) and digital unit (DU) configuration to access unit (AU), DU, and central unit (CU) configuration. In the AU, a physical layer is added to the existing RU, and the time division duplex (TDD) communication method is applied. Additionally, due to the cloudification of DU, CU is newly introduced and takes a role of orchestration. Along with changes in wireless networks, core networks have changed to a decentralized configuration to separate CP and UP and minimize traffic transmission delay. Therefore, a decentralized security configuration needs to be considered for core network security rather than existing centralized configuration. Security requirements for 5G mobile communication networks that require super-connection, ultra-fast, and ultra-low latency are expanding significantly [11].

Table 2 shows the commercialization schedule of respective network configurations, and the scope of this paper covers NSA only, as SA configuration is not actively commercialized yet.

Followed by the description above, we would like to describe attachment procedure, a procedure for NSA terminal to access the 5G network.

### 2.2. 5G NSA Attachment Procedure

**Figure 2:**
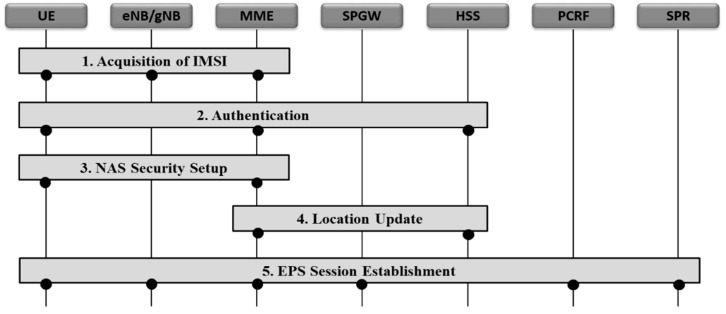
NSA Attachment procedure of 5G NSA.

As shown in Figure 2, the attachment procedure of a 5G NSA terminal consists of a total of five steps [13]. The first is the process of obtaining international mobile subscriber identity (IMSI) from the network, and the second is the process of authenticating the subscriber. The third is the process of security setup for NAS message between the terminal and MME, and the fourth is the process of location update between the MME and HSS. Finally, the fifth step is the process of evolved packet system (EPS) session establishment to create data session for the terminal. Next is detailed descriptions for NAS security setup in the 3rd step and EPS session establishment in the 5th step, which are key steps utilized in security threats that will be described subsequently.

#### 2.2.1. 5G NSA NAS Security Setup Procedure

**Figure 3:**
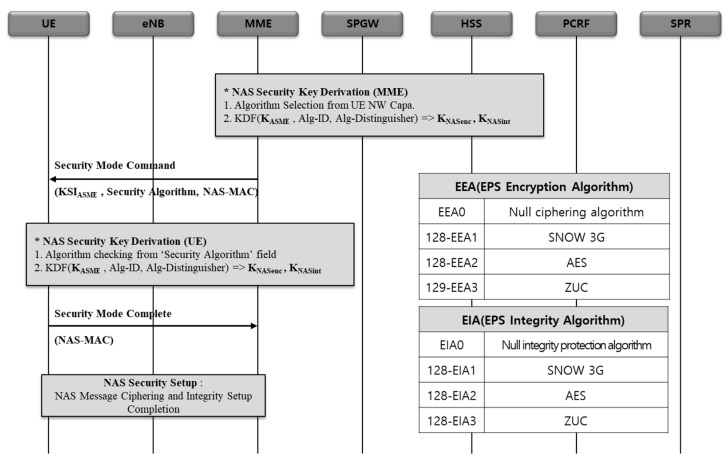
NAS security setup procedure of 5G NSA.

As shown in Figure 3, the NAS Security Setup process is a preparatory task to deliver signal messages safely between the terminal and the MME on a wireless link. It performs an integrity check and ciphering function for NAS signaling messages. While an integrity check is defined as mandatory in 3GPP TS. 33.401, ciphering is defined as an option [14].

When a terminal (UE) sends a network access request (attach request) message (non-integrity, non-ciphering) to a 5G network, the value set in the UE Network Capability field in that message represents the type of ciphering and integrity protection algorithm supported by the terminal. Among EPS encryption algorithm 0 (EEA0) (Null), 128-EEA1 (SNOW3G algorithm), 128-EEA2 (AES algorithm), and 128-EEA3 (ZUC algorithm), an algorithm that be supported by the UE is set for ciphering algorithm. As shown in Figure 3, among EPS integrity algorithm 0 (EIA0) (Null), 128-EIA1 (SNOW3G algorithm), 128-EIA2 (AES algorithm), and 128-EIA3 (ZUC algorithm), an algorithm that be supported by the UE is set for integrity protection algorithm [15].

Based on the value set in the UE Network Capability field in the terminal request message, MME selects ciphering and integrity algorithms that will be applied to the NAS message. In response to the request from UE, MME sends NAS security mode command message (integrity, non-ciphering) that includes replayed UE security capability (a field set by copying the value as it is set in the UE network capability field in the Attach Request message of the UE), NAS ciphering algorithm and NAS integrity protection algorithm selected by the MME, and other key information [14].

The UE recognizes the ciphering and integrity algorithm selected by the MME, and sends NAS security mode complete message (integrity, ciphering) to the MME through verification process.

The MME verifies the integrity of NAS security mode complete message, decodes the message, and then sends an attach accept message that allows for the UE to access the network to the UE. Subsequently, access stratum (AS) security setting process is carried out between the UE and a base station [16].

#### 2.2.2. 5G NSA EPS Session Establishment Procedure

5G NSA EPS session establishment procedure consists largely of three procedures: (1) S5 GTP-U tunnel creation, (2) data radio bearer (DRB) creation between the terminal and eNB, and S1 GTP-U tunnel creation between eNB and SGW, (3) DRB creation between the terminal and gNB, and S1 GTP-U tunnel creation between gNB and SGW [17]. The reason why DRB and S1 GTP-U tunnels are created repeatedly is that NSA is configured with E-UTRA new radio-dual connectivity (EN-DC). The purpose of this is to form eNB and bearer first for master base-station, followed by gNB and bearer for secondary base-station.

**Figure 4:**
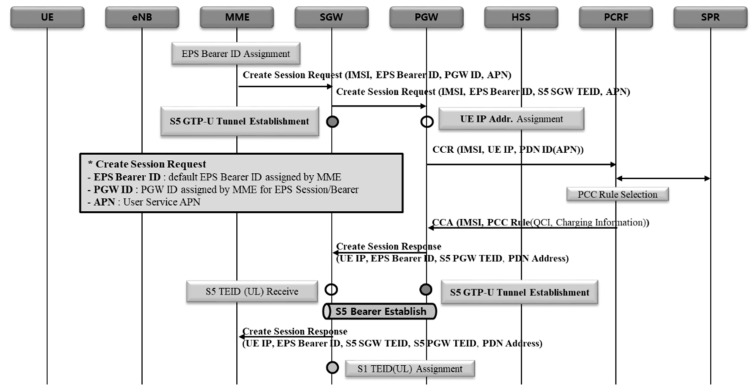
S5 GTP-U tunnel creation procedure.

**Figure 5:**
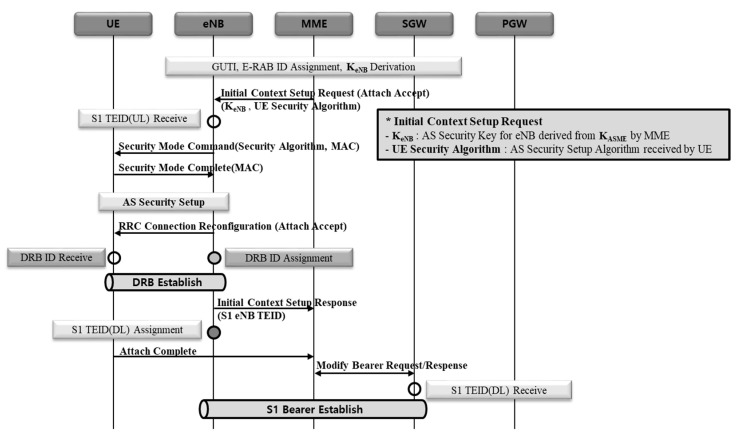
eNB DRB and S1 GTP-U tunnel creation.

**Figure 6:**
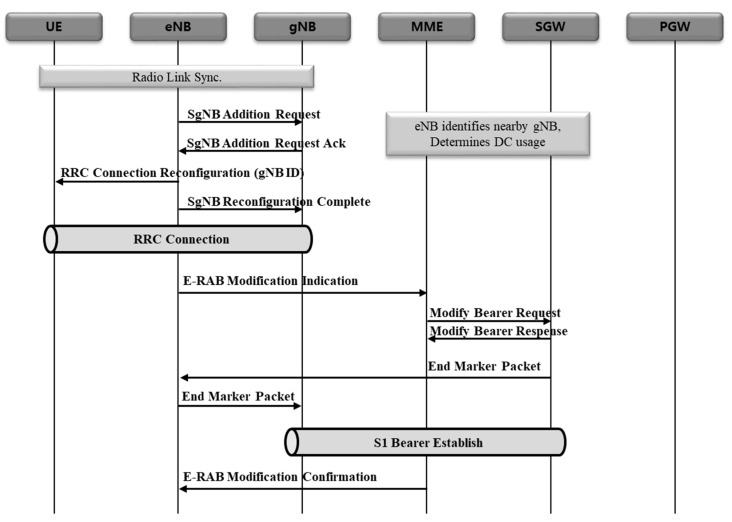
gNB DRB and S1 GTP-U tunnel creation.

1. (S5 GTP-U tunnel creation) Figure 4 shows the procedure to create S5 GTP-U tunnel represented in a sequence diagram. UE IP is allocated in PGW first, followed by S5 GTP-U tunnel creation between SGW and PGW.
2. (eNB DBR and S1 GTP-U tunnel creation) Figure 5 shows the procedure to create eNB DRB and S1 GTP-U tunnels represented in a sequence diagram. Where, DRB is formed between a terminal and eNB, and then S1 bearer is formed between eNB and SGW.
3. (gNB DRB and S1 GTP-U tunnel creation) Figure 6 shows the procedure to create gNB DRB and S1 GTP-U tunnels represented in a sequence diagram. Finally, DRB is formed between the terminal and gNB, and then S1 bearer is created between gNB and SGW [18].

## 3. Related Work

Ghada Arfaoui et al. cover new cases of use from new business environments provided by security architecture for 5G networks and related security issues arising from the use of new technologies. The authors present the design goal of security architecture for 5G and provide the full details of safe 5G networks using the defined architecture. Secondly, the authors elaborate on the concept and the components of the architecture used. However, their research simply applies the proposed security architecture to cases involving IoT use for smart cities and shows the feasibility of application without specific details on how to test the security issues presented and reproduce the threats on the actual network [19].

In order to analyze security vulnerabilities in LTE CP, Hong-il Kim et al. introduced a three-step procedure: extracting security properties, creating test cases, and classifying problematic behaviors. For extracting security properties, they defined three security properties that networks and mobile devices need to comply during LTE CP process. The first property is to ensure that there is no unexpected input in the received entity when the counterpart sends plain message created in the initial procedure. The second property is to certify whether the respondents can handle a message unencapsulated by the security header. The last property is to ensure that the security procedure complies with 3GPP standards. The risk was demonstrated by creating test cases through the security property and testing them on a actual network using universal software radio peripheral (USRP) to reproduce the threats. However, only the risks researched through the security property can be simply verified because specific countermeasures are not provided in that paper [5].

Merlin Chlosta et al. conducted vulnerability inspection on 12 LTE networks in four European countries. They assumed that if the network is set without ciphering and integrity, the network would select an algorithm from the list of selected algorithms in the security mode command message or reject the access. However, they found behaviors that did not comply with the standard in the experiment. That paper presents actual test results for European mobile carriers and addresses 3GPP standards for integrity and ciphering, but it does not provide relevant countermeasures specifically in terms of technology or standardization [6].

Ijaz Ahmad et al. listed security threats for all technical areas available in 5G and presented security solutions for each area. They divided the areas broadly into 4 areas: SDN, network functions virtualization (NFV), mobile cloud and mobile edge computing (MEC), and privacy, and organized security challenges based on target points and network elements. However, they neither mentioned how to attack security challenges in actual networks, nor described solutions specifically in the 4 areas [20].

Existing studies were limited due to focusing only on security threats and suggesting only rough way of coping with the security threats. Accordingly, this paper specifically describes how to apply and test security threats on actual networks. This paper also differs from existing studies by specifically presenting how to mitigate each of the security threats on an actual network.

## 4. Security Challenges

Unlike the SA configuration, the fact that NSA configures the core network with LTE-based EPC, and uses eNB means inherent LTE security threats from a security point of view. Thus, LTE-based security threats can be applied as they are in order to check security vulnerabilities for 5G NSA networks. We’d like to discuss security threats and countermeasures against the NSA networks in this paper.

**Figure 7:**
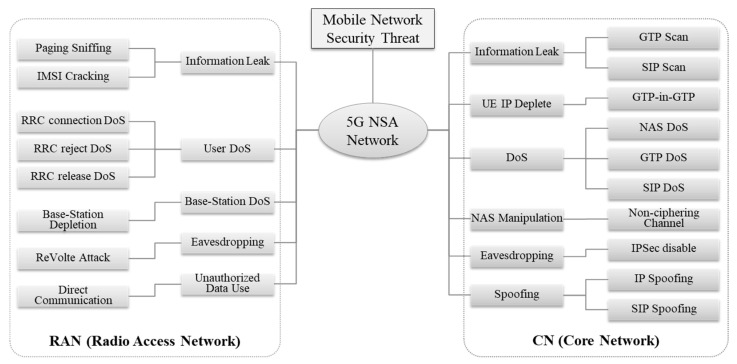
5G NSA attack tree.

Most security threats in 5G NSA networks exploit LTE vulnerabilities, including information leak, target-type user denial of service (DoS), target-type network device DoS, voice eavesdropping, and unauthorized data use. If you create an attack tree as shown in Figure 7, you can distinguish wireless network security threats from core network security threats.

### 4.1. 5 Types of RAN Security Threats

1. **Type.R1. Information Leak**: Information leak involves threats such as paging sniffing and IMSI cracking. Paging sniffing is a method of passive scanning all kinds of information by exploiting the broadcasting of paging messages transmitted from wireless base stations to terminals. The attacker can install a software defined radio (SDR) device that can receive radio frequency (RF) signals near the target victim and find out the victim’s SAE-temporary mobile subscriber identity (S-TMSI) or paging cycle. Additionally, the attacker can calculate paging frame index (PFI) using the identified paging cycle and reduce the number of candidates for the victim’s IMSI to 8 at most using the calculated PFI. The attacker sends IMSI paging where the 8 IMSI candidates are inserted to the victim and observes the responses to intercept the victim’s IMSI [21].
2. **Type.R2. User DoS**: Radio resource control (RRC) connection DoS, RRC reject DoS, and RRC release DoS are available for target-type user DoS. Among them, RRC connection DoS is a security threat using the victim’s S-TMSI that was found through the aforementioned information leak attack. Unlike the core network, wireless base stations do not have authentication procedures for terminals, which can be exploited to continuously interfere with the victim’s wireless access. The attacker inserts the victim’s S-TMSI value into the RRC connection request message used in wireless access by the terminal, and sends the message to the base-station accessed by the victim. On this occasion, the base-station disconnects the victim’s RRC connection but makes RRC connection with the attacker, which is disconnected in the course of attempting security setup from the attacker’s terminal. After this, it would not be a problem if the victim’s terminal made a normal RRC connection successfully, but the attacker keeps sending the manipulated RRC connection request message and the victim will not be able to get the service continuously [5].
3. **Type.R3. Base-Station DoS**: A typical case of target-type network device DoS in a wireless network is a wireless base-station resource depletion. At the time of the first RRC connection, the terminal performs Random Access that creates a random ID and sends RRC connection request. An attacker can exploit this to attempt RRC connection and transfer even NAS attach request using the victim’s IMSI. When requesting authentication and waiting for the response in the core network, the attacker can perform Random Access again, repeat the above process, and keep increasing the number of RRC connection in the base-station [5].
4. **Type.R4. Eavesdropping**: In principle, eavesdropping on wireless networks is impossible due to AS security settings between terminals and base stations. However, there can be a case that involves extracting AS Security Keystream and decoding. Voice traffic in mobile communication consists of real-time transport protocol (RTP) protocol, which is delivered using a voice bearer, unlike a data bearer in wireless sections. Because quality of service (QoS) must be guaranteed, a voice bearer is not a default bearer but a dedicated bearer where a separated QoS class identifier (QCI) is applied. A total of 4 elements are used when creating keystream for ciphering in AS security procedure: count, direction, length, and bearer ID. Three of these four elements, except bearer ID, do not act as critical variables in creating the keystream. In particular, bearer ID (DRB ID) is allocated when a base-station creates voice bearer, and a manufacturer’s base-station may have a problem of allocating the same DRB ID within the same RRC connection. In order to exploit this problem, an attacker keeps the victims’ ciphered voice communication using a sniffer. Not long after the victims’ calls are ended, the attacker attempts to make a voice call to one of the victims, and keeps the plan-text and cyber-text of the call using a sniffer when the call is made. On this occasion, the attacker applies the plane-text and ciphered-text of the second call to XOR logic to extract keystream, and the extracted keystream can be used to decode the first call because these calls was made in the same RRC connection and used the same DRB ID. 3GPP TS 33.401 recommends the use of different DRB IDs in other bearers to prevent a DRB ID from being reused in a base station [22].
5. **Type.R5. Unauthorized Data Use**: Two bearers, a default bearer and a dedicated bearer, created in a terminal have to be used for permitted purposes, but the attacker can use them differently from the original purpose to use the data without permission. It is possible to have data communication between terminals without paying any communication fee using a dedicated bearer. Especially, caller spoofing is also possible by utilizing direct communication properly [23].

### 4.2. 6 Types of Core Network Security Threats

1. **Type.C1. Information Leak**: Information on 5G NSA core networks can be largely divided into information on EPC equipment to process the data and information on IMS equipment to provide various services. Because EPC equipment communicates using GTP protocol and IMS equipment communicates using session initiation protocol (SIP) protocol, the attacker can select a protocol suitable for the desired information. GTP protocol is divided into GTP-C used between core network equipment, and GTP-U that delivers data traffic in the user terminal through a tunnel between the base station and PGW. In order to find out the IP information of the EPC equipment, the attacker can use a packet injection method that loads an echo request, GTP-C message for health check between core network equipment, on the data payload to send. When running Android debug bridge (ADB) command in Android terminal using a program called Packit, a packet is created, and when sending the packet to the IP band identified through Tracert in tethering status, the GTP-C packet is injected and transmitted to the mobile communication network. PGW checks this and sends echo response, where the attacker can identify that the source IP of that message is PGW IP [24].
2. **Type.C2. IP Depletion**: The packet injection method described earlier to provoke information leak threat is called GTP-in-GTP, and the attacker can deplete IP Pools allocated to terminals in the core network through the same method. While GTP-C echo request that plays a role of ping is used to acquire IP for core network equipment, GTP-C Create Session Request is injected and sent to the core network to allocate the IP to the terminal. The attacker can increase the terminal number in the create session request sequentially so that PGW allocates multiple IPs. If PGW allocates all of available IPs, create session requests from normal terminals would be rejected, and all of terminals accessing that core network could not communicate [24].
3. **Type.C3. DoS**: An attacker can send an attach-request message continuously to access the 5G NSA network by configuring multiple terminals as botnets and repeating airplane mode on/off. This may cause excessive traffic load on a certain mobile carrier’s core network. One attach request can create maximum eight GTP-C messages, which brings 8 times the amount of traffic to the CN function in the core network in proportion to one malicious manipulation done by the attacker [25].
4. **Type.C4. NAS Manipulation**: Of NAS protocol messages for signaling between terminals and core network, attach-request messages used in the initial attaching step do not have their ciphering or integrity guaranteed. Therefore, an attacker can install a rogue base-station near the victim to steal and manipulate those messages. In particular, an attach request-message has UE network capability field that can set ciphering or integrity for all data received or transmitted by the terminal. An attacker can manipulate values in EEA that is a field to transmit ciphering algorithm selected by the terminal, and EIA that is a field to transmit integrity verification algorithm selected by the terminal, within the UE network capability field. 3GPP technical specification (TS.) 33.401 defines the essential use of integrity verification algorithm in terminals but defines the selective use of ciphering algorithm. In fact, the test results conducted by Ruhr university in Germany in 2019 on five European countries and 12 carriers showed that four of 12 carriers do not even allow the use of integrity that must be used [6].
5. **Type.C5. Eavesdropping**: Voice communication on a 5G network uses IMS network and initiates session through SIP protocol according to 3GPP standard. Therefore, security in SIP protocol is very important and done mainly through internet protocol security (IPSec) security associations (SAs). However, IPSec SAs is also selectively done by 5G network operators, and supporting voice over LTE (VoLTE) does not mean supporting all IPSec because of its significant impact on the terminal performance. The Samsung Galaxy S10 model, a recently released 5G terminal, also supports IPSec, but there is a problem in which the setting in question can be turned off through a hidden menu. If an attacker can remotely access the victim’s hidden menu and change the IPSec setting, the victim’s call will communicate without ciphering. If EEA field is changed through NAS manipulation described above and NAS ciphering algorithm is not used, wireless communication in AS section is non-ciphered either. In this situation, an attacker can sniff wireless traffic in the form of man in the middle (MitM) and eavesdrop on the unencrypted victim’s voice traffic as it is [26].
6. **Type.C6. Spoofing**: IP spoofing is a typical network attack. If an attacker changes the IP of data traffic transmitted from every 5G network to the victim’s IP and sends the data traffic, its responses are all delivered to the victim, which can cause invalid charging and even DoS. Additionally, SIP or MMS spoofing can be abused for voice phishing. When the “from” header that indicates the outgoing number in the SIP packet header is falsified, the incoming terminal displays that falsified number [24,27].

## 5. Security Threat Test Cases

We created 15 test items to analyze vulnerabilities in 5G NAS network. These items were largely categorized and organized by planes, and developed into 7 items in CP and 8 items in UP.

### 5.1. RAN Test Cases

First, Table 3 summarizes the 3 CP test cases in RAN section.

### 5.2. CN Test Cases

12 cases that can be tested in the CN section can be divided into CP and UP as shown in Table 4 and Table 5.

If you analyze where the developed test cases belong to which security challenges in Section 4, you can have mapping as shown in Table 6. If there is no test case, existing studies can be checked additionally.

## 6. Test Tool Implementation and Test Environments

### 6.1. Tool Development

**Figure 8:**
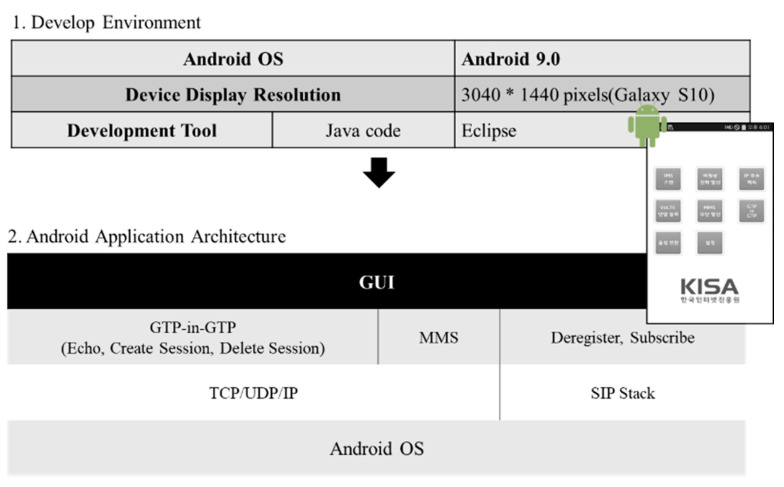
Android application architecture for 5G security threat assessment.

Two types of tools have been developed to analyze vulnerabilities in the 5G NSA network. The first is a tool that can operate on application above layer 4 based on open system interconnection (OSI) 7 Layer. The operating system was developed in Android application package (APK) format file to run on Android 9.0 or higher, so that it can be actually installed and used in 5G terminal. The detailed development environment is as shown in Figure 8. The source code of the tool was written in the Java using Eclipse. If it is distributed through platforms such as the Github in the future, anyone can easily customize it.

A total 8 of attack types are loaded for Android application attack tools, and a separate protocol stack was constructed for each type. In addition, a packet capture function was developed utilizing packet capture (PCAP) library so as to check packets transmitted or received by the terminal. Attack packets created with this tool are transmitted on UP, capsulated with GTP-U in a base-station, and transmitted to the core network. Therefore, detection or blocking is very difficult unless GTP detection system related to the user data is installed in the communication network.

GTP-in-GTP function can inject echo request/response, create session request/response, and delete session request/response messages modulated by mobile carriers into data payload and transmit the messages.

**Figure 9:**
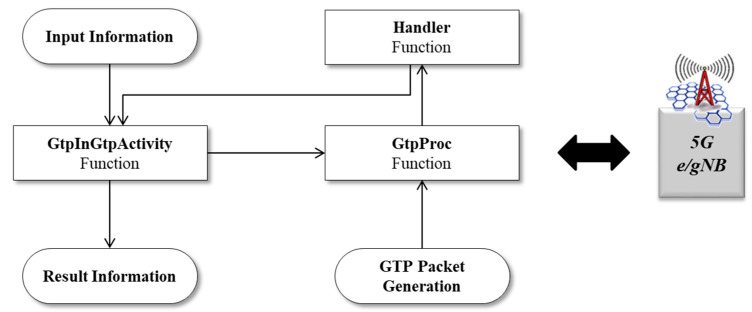
Structure of Android application for 5G security threat assessment.

The structure of the Android application attack tool is as shown in Figure 9.

**Figure 10:**
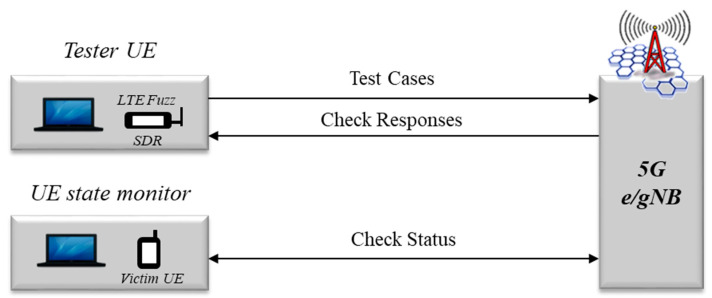
Testing procedure of LTE Fuzz for 5G security threat assessment.

As shown in Figure 10, the second tool to analyze vulnerability, is a tool that transmits messages modulated on CP such as RRC and NAS, and triggers test cases according to the attach or authentication procedure. It can operate on layer 3 based on OSI 7 Layer and has been developed to run in a virtual terminal environment such as USRP by applying LTE Fuzz tools [5]. Additionally, we utilized SDR and srsLTE, an open source mobile communication modem, to send the created test cases, and checked the status changes of 5G terminal affected by the test cases by using the open source terminal diagnostic tool, SCAT. In particular, we established an independent dynamic analysis environment by separating internal information including logs and debugging messages of test tools in consideration of the diversity of 5G NSA network equipment for each manufacturer, and implemented dynamic analysis to analyze vulnerabilities based on the status change of the terminal.

### 6.2. Test Environment

**Figure 11:**
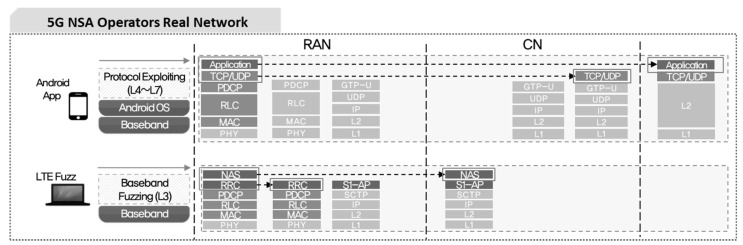
Testing environment for 5G security threat assessment.

The test was carried out on a total of three mobile carriers. Each mobile carrier uses equipment from many manufacturers, but the test can be done on base-station and core network equipment from limited manufacturers due to some issues such as manpower support. As shown in Figure 11, we configured an environment identical with actual commercial network and carried out the test using test universal subscriber identify modules (USIMs) and terminals registered in the test network of each mobile carrier. Finally, we analyzed terminal test logs and packets using OPTis-S, a commercial charged diagnostic monitor (DM) program from Innowireless corporation.

## 7. Test Results and Mitigation in Real Networks

**Test Results.** Different vulnerabilities were found for each mobile carrier, which was verified due to differences in software packages depending on equipment manufacturers. Table 7 shows vulnerabilities found for each mobile carrier, where “O” means vulnerable.

**Measures.** When counting the same vulnerabilities found in different mobile carriers as one, the total number of problems found is three in RAN section and five in CN section. As shown in Table 8, measures could be taken with equipment SW patch or changing configuration setting for a total of 5 cases, while standardization or a countermeasure system is required for the remaining three cases. As a result, the main challenges in this paper are RRC Connection DoS, NAS Ciphering Spoofing, and SIP Spoofing, which cannot be taken with SW patch or changing config-uration of network components. The standardization is underway to address these threats, but this process takes much time to address.

Additionally, although the threat of UE IP depletion was not found in an actual network, it is the most basic attack that can be tried on 5G NSA core network with EPC scanning. Thus, a detection method through IDS or IPS was suggested rather than standardization.

## 8. Countermeasures and Future Work

This section explains countermeasures for vulnerabilities that require additional security measures other than vulnerabilities in implementation flaw supplemented by mobile carriers’ equipment patches.

There can be many ways of responding to these threats. However, as mentioned above, we can consider approaching countermeasures against 5G security threats in a total of two levels: standardization of the security guide and detection through intrusion detection system (IDS) or intrusion prevention system (IPS).

### 8.1. Countermeasures through Standardization

**User DoS (RRC connection DoS).** The root cause of the RRC connection DoS threat is that RRC connection request, a message transmitted when an user terminal accesses the network, is transmitted in plain text, and the message includes TMSI, a temporary identification information of the user terminal. In order to respond to this, the forgery of RRC messages must be verified at the base-station level, which is not easy to define in 3GPP. Additionally, blocking attackers from finding out the temporary identification information of a specific user can be a way to verify, which is not easy either because there are too many known methods. Utilizing temporary identification information in RRC Connection request is to prevent privacy invasion caused by leakage and abuse of IMSI which is subscriber identification information within USIM, but attackers can intercept RRC Connection request messages sent in plain text and easily identify TMSI, which is temporary identification information. Through this, the attacker can create and transmit a modulated RRC Connection request message so that the base-station mistakes the message for one sent by the victim UE. In the core network, temporary identification information is created with a certain time interval according to specific rules based on IMSI, and even if the TMSI is changed, the attacker can identify the changed TMSI and create an attack message again. When receiving the modulated RRC connection request, the base stations cancel the connection to the existing victim’s terminal without checking modulation status and allows access to the attacker’s terminal. In such situation, if the base-station does not disconnect from exiting victim’s terminal or keeps the connection for a certain period of time, it can reduce the DoS threat of the victim. Because the attacker’s terminal fails authentication after the RRC Connection, maintaining the connection to the existing terminal only during the period when the attacker’s terminal sends the RRC connection request and the authentication fails may not significantly affect the performance of the base station.

The above countermeasures are matters of implementation in the base station, and reflecting them in 3GPP TS. 33.401 is a matter to be reviewed carefully considering the scope or depth of the standards. On the other hand, it would be more feasible to present relevant countermeasures as security guidelines for 5G base-stations and propose them as related international standards such as ITU-T. In the future, relevant standardization will be promoted so that mobile carriers providing 5G services can use relevant countermeasures as a reference guide to enhance their security.

**NAS Manipulation (NAS ciphering spoofing).** 3GPP 5G standard supports both integrity verification and cipher communication to reinforce NAS protocol security between terminals and 5G core networks. However, because cipher communication is not mandatory but optional function among NAS protocol security functions, there are some cases of security functions not being used according to the policy of the country or the mobile carrier (3GPP TS. 33.401). In addition to emergency calls, some countries or carriers may not use ciphering capabilities provided by 5G standards in terms of security.

Additionally, the 5G standard does not define mutual authentication between terminals (UE) and 5G networks or the integrity verification function of initial messages exchanged before ciphering, which is based on underlying trust assuming that the initial messages are not modulated. This problem arises from a vulnerability that does not confirm the forgery of the first access request message (attach request) to 5G network sent by UE. A malicious attacker infiltrates between a victim’s UE and a base-station, manipulates an access request message including a request for ciphering and integrity verification normally sent from the terminal into ciphering disabled and integrity unverified access request message, and sends it to a normal base-station.

5G core network equipment (MME) forms a non-ciphering channel with the UE according to the ciphering disable request which is manipulated message differ from the original message sent by the UE, even though ciphering enable is the default. This problem occurs because the 3GPP standard does not incorporate integrity authentication procedure to check the forgery of the initial message prior to mutual authentication stage, which results in non-ciphering communication caused by manipulated non-ciphering and integrity-unverified UE messages.

Two threats can arise from this, and the first one is the manipulation of message content. There are vulnerabilities or risks where malicious attackers can modulate the messages sent from a victim’s terminal as they wish. This is because the standard does not define any integrity verification procedure to check whether the access request message sent by a terminal during the first access to the communication network has been manipulated or not. The second is eavesdropping on communications. Due to a ciphering disable request within the modulated initial access message, communication between the terminal and 5G network will be formed as a non-ciphering channel, which allows all wireless communications to be intercepted by a malicious attacker, and personal information and location information contained in the exchanged messages to be exposed.

An attacker can disable ciphering settings between the victim’s terminal and the core network using a fake base-station to use the victim’s data or tap the contents of the message. We implemented an algorithm to detect this and conducted detection performance tests. By analyzing the ciphering fields in NAS protocol messages between terminals and core networks, it is possible to set ciphering bypass channels or determine whether they are non-standard terminals. First, arranging the ciphering bypass channel to detect cases where the EEA in the UE network capability field is set as full-cipher (EEA0) when a terminal accesses 5G NSA network. Secondly, although the change of UE Capability in Replayed UE network capability settings on the terminal can be confirmed for non-standard terminals, a case that completes NAS security complete step without rejecting NAS security complete step can be detected. As a result, a plan is in place to standardize guides for a security system to detect these NAS non-ciphering channels.

**Eavesdropping (SIP spoofing).** Because eavesdropping caused by SIP spoofing is possible when the IPSec is lifted, it is necessary to guide voice communication ciphering settings between terminals and the 5G network to be managed by the mobile carrier’s network instead of being processed according to the terminal function (selective application is required in the network for IPSec unsupportive terminals). If the IPSec setting of the 5G voice service is determined by the terminal function, malicious users may attempt multiple attacks by exploiting their terminal settings. Additionally, we need efforts for raising awareness to publicize the risk of leaking the details of communication when attackers maliciously modulate messages and engage in non-ciphering communication between the terminal and 5G network. The applicable section for IPSec is defined as local policy in 3GPP, but a review is necessary regarding making it mandatory at the 3GPP standard level. Accordingly, we tried a vulnerability report on the above issues to 3GPP and discussed these issues through a collaborative mobile carrier at SA3 e-meeting in November 2020. Although it is difficult to induce any change in the standards right now, improvements need to be suggested consistently to combat 5G security threats.

### 8.2. Technical Countermeasures

This subsection describes technical countermeasures against 5G security threats. First, a system configuration for response will be presented, followed by an algorithm that can detect the threats.

**Figure 12:**
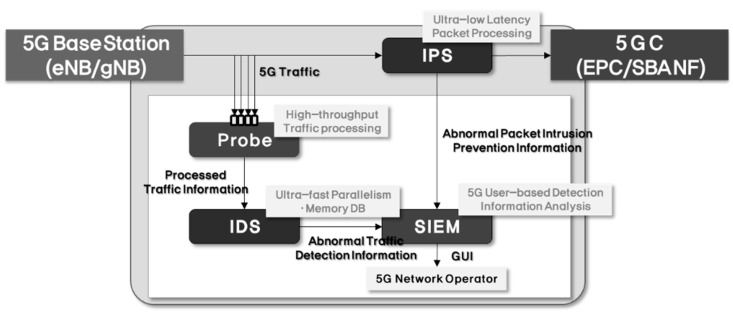
System architecture for 5G security threat countermeasure.

**System Architecture for Countermeasure**. As shown in Figure 12, Probe and IDS are configured with traffic-based security threat detection system and IPS is configured with packet-based security threat detection system. The detection information generated from each system is monitored by the security information and event management (SIEM) and relayed to the operators.

**Information Leak (GTP-in-GTP: echo request).** Following Algorithm 1 is the detection algorithm for traffic that scans MME, SGW or PGW IP through the GTP-in-GTP packet injection attack in the countermeasure architecture mentioned above.
**Algorithm 1. Scanning traffic detection algorithm.****Data:** (1) Value of Identification a IP Packet N(P)
    (2) Destination Port of the IP Packet DP(P), Destination Port of Payload in the IP Packet DP(Pin)
    (3) two protocols Gc, Gu, and the Destination Ports of these two protocols DP(Gc), DP(Gu)
    (4) Value of Upper 2Bytes of Payload in the IP Packet UB(Pin)
    (5) Flags Header’s values of GTP-C Echo Request messages F(Gce)
    (6) Payload’s Length in the IP Packet L(Pin), Value of Message Length Header of Payload in the IP Packet ML(Pin)
**Result:** Scanning Detection Result of the IP Packet from 5G UE to 5G EPC SD(P)
*DP* (*Gc*) = 2123; // GTP-C Port Number
*DP* (*Gu*) = 2152; // GTP-U Port Number
*F* (*Gce*) = 4001; // Flag if *DP* (*P*) == *DP* (*Gu*)&&*DP* (*Pin*) == *DP* (*Gc*) then
  UB(Pin) = Value of Upper 2Bytes of Payload;
  if *UB*(*Pin*) == *F* (*Gce*) then
    L(Pin) = Payload’s Lengths;
    ML(Pin) = Value of Message Length Header of Payload;
    SD(P) = 0;
    if *L*(*Pin*) == 21&&*ML*(*Pin*) == 9 then
      SD(P) = 1;
    else
    end if
  else
  end if
else end if
return SD(P) and N(P);

**IP Depletion (random create session request).** Following Algorithm 2 is the detection algorithm for Resource Exhaustion traffic that maliciously induce IP allocations carried out by PGW in the countermeasure architecture mentioned above.
**Algorithm 2. Resource exhaustion traffic detection algorithm.****Data:** (1) Value of Identification a IP Packet N(P)
    (2) Destination Port of the IP Packet DP(P), Destination Port of Payload in the IP Packet DP(Pin)
    (3) two protocols Gc, Gu, and the Destination Ports of these two protocols DP(Gc), DP(Gu)
    (4) Value of Upper 2Bytes of Payload in the IP Packet UB(Pin), Value of Upper 12th 1Byte of Payload in the IP Packet UB12(Pin)
    (5) Flags Header’s value of GTP-C Create Session Request messages F(Gcc), Spare Header’s value of GTP-C Create Session Request message S(Gcc)
    (6) Payload’s Length in the IP Packet L(Pin), Value of Message Length Header of Payload
    in the IP Packet ML(Pin), Message Length of Create Session Request message in the IP Packet MLc(Pin)
**Result:** Resource Exhaustion Detection Result of the IP Packet
    from 5G UE to 5G EPC RED(P) *DP* (*Gc*) = 2123; // GTP-C Port Number *DP* (*Gu*) = 2152; // GTP-U Port Number
*F* (*Gcc*) = 4820;
*S* (*Gcc*) = 0;
if *DP* (*P*) == *DP* (*Gu*)&&*DP* (*Pin*) == *DP* (*Gc*) then
  UB(Pin) = Value of Upper 2Bytes of Payload;  UB12(Pin) = Value of Upper 12th 1Byte of Payload;  if *UB*(*Pin*) == *F* (*Gcc*)&&*UB*12(*Pin*) == *S* (*Gcc*) then    L(Pin) = Payload’s Lengths;
    ML(Pin) = Value of Message Length Header of Payload;
    MLc(Pin) = L(Pin) − 12;    RED(P) = 0;
    if *L*(*Pin*) < 280&&*L*(*Pin*) > 200&&*ML*(*Pin*) == *MLc* (*Pin*) then
      RED(P) = 1;
    else
    end if
  else
  end if
else
end if
return RED(P) and N(P);

## 9. Concluding Remarks

Through this study, we have identified various security threats that may arise in the 5G NSA network, verified them on actual network, and suggested ways to enhance security. In addition, we showed persistent vulnerabilities in existing mobile network system through investigation for recent 5G studies, and looked at the need for new security techniques rather than traditional security methods and related research.

It seems that the testing and verification of 5G networks provided by service providers will be needed in the future, and research on verification and defense measures against previously proposed attacks and newly proposed attacks will be needed as well. In addition, 5G service operators will build a 5G SA environment that has previously not existed and provide customers with new and convenient services that utilize the advantages. However, it is also necessary to think at least once about what security threats exist in new services and consider related countermeasures before launching these services.

We hope that this work would help people use safe 5G services. Subsequently, we will study SA structures and threats in B5G environments through follow-up research.

## Figures and Tables

**Table 1:** Wireless Infrastructure Revenue Forecast, Worldwide, 2018–2021 (Millions of Dollars).

Segment	2018	2019	2020	2021
5G	612.9	2211.4	4176.0	6805.6
2G	1503.1	697.5	406.5	285.2
3G	5578.4	3694.0	2464.3	1588.0
LTE and 4G	20,454.7	19,322.4	18,278.2	16,352.7
Small cells	4785.6	5378.4	5858.1	6473.1
Mobile Core	4599.0	4621.0	4787.3	5009.5
Total	37,533.6	35,924.7	35,970.5	36,484.1

**Table 2:** 4G and 5G mobile technologies comparison [12].

Segment		4G	5G Non-StandAlone	5G StandAlone
Launching date		11 July	18 December	20 August (USA) ~ongoing
Peak data rate (Downlink)		1 Gbps	20 Gbps	20 Gbps
Latency		10 ms	1~10 ms	1 ms
RAN (Radio Access Network)	User Equipment	Smart phone	Smart phone, Internet of Things, Cyber Physical System	Internet of Everything, Autonomous Vehicle
	RAN type	Single RAN (eNB)	Hybrid RAN (eNB/gNB)	SDRAN (gNB)
	Control protocol	RRC, NAS	RRC, NAS	RRC, NAS
	User protocol	PDCP	PDCP	PDCP
CN (Core Network)	CN type	Centralized (EPC)	Centralized (5G Enabled EPC)	Distributed (5GC)
	Control protocol	GTP-C	GTP-C	HTTP/2
	User protocol	GTP-U	GTP-U	GTP-U

**Table 3:** RAN security threat test cases.

Index	Test Case	Vulnerability Description
TC. R1	RRC connection DoS	According to the standard, it is defined not to verify subscriber ID in base-stations, so access is allowed when sending RRC connection request with a victim’s ID.
TC. R2	RRC security mode command	There is threat of avoiding authentication when a base station ignores authentication value (MAC) from the terminal for received RRC Security mode command and sends RRC Security mode complete, thus it is processed normally in the base-station.
TC. R3	RRC connection reconfiguration	There is a threat of avoiding authentication when a base station ignores authentication value (MAC) from the terminal for received RRC Connection reconfiguration and sends RRC Connection reconfiguration complete, thus it is processed normally in the base-station.

**Table 4:** CN CP security threat test cases.

Index	Test Case	Vulnerability Description
TC.C-CP1	NAS Integrity Spoofing [6]	Avoiding the verification of NAS message integrity by changing the EIA field in UE Network Capability of victim’s attach request to 0.
TC.C-CP2	NAS Ciphering Spoofing [6]	There is a threat of eavesdropping when ciphering is not used by changing the EEA field in UE Network Capability of victim’s attach request to 0.
TC.C-CP3	NAS Security mode command [5]	There is threat of avoiding authentication when MME ignores authentication value (MAC) from the terminal for received Security mode command and sends Security mode complete, thus it is processed normally in the MME.
TC.C-CP4	NAS Attach accept [5]	There is threat of avoiding authentication, when MME ignores authentication value (MAC) from the terminal for received Attach Accept and sends attach mode complete, thus it is processed normally in the MME.

**Table 5:** CN UP security threat test cases.

Index	Test Case	Vulnerability Description
TC.C-UP1	EPC scanning [24]	EPC equipment IP can be identified through the response message received after injecting GTP-C echo request into the user data and sending it to CN.
TC.C-UP2	UE IP depletion [24]	Causing IP resource depletion that can be allocated by PGW when injecting Create Session Request that contains random NISIDN into the user data and transmitting the request.
TC.C-UP3	Targeted create session request [24]	Causing DoS by newly allocating the victim’s IP when injecting Create Session Request that contains the victim’s NISIDN into the user data and transmitting the request.
TC.C-UP4	Delete Session request [24]	Causing DoS by deleting the victim’s GTP session when injecting Delete Session Request that contains the victim’s NISIDN into the user data and transmitting the request.
TC.C-UP5	SIP de-register request [27]	Causing voice service DoS by deleting the victim’s SIP Registration when transmitting SIP de-Register Request that contains the victim’s MSISND.
TC.C-UP6	SIP bye request [27]	Causing voice communication termination by deleting the victim’s SIP Invite when transmitting SIP Bye Request that contains the victim’s MSISND.
TC.C-UP7	SIP message request [27]	Causing SMS phishing with the outgoing number when transmitting SIP Message Request that contains the victim’s MSISND.
TC.C-UP8	MMS request [27]	Causing MMS phishing with the outgoing number when transmitting MMS Request that contains the victim’s MSISND.

**Table 6:** Security challenges and test cases map.

Test Cases	Security Challenge Types										
	RAN					CN					
	R1	R2	R3	R4	R5	C1	C2	C3	C4	C5	C6
TC. R1	-	√	√	-	-	-	-	-	-	-	-
TC. R2	√	-	-	√	√	-	-	-	-	-	-
TC. R3	√	-	-	√	√	-	-	-	-	-	-
TC.C-CP1	-	-	-	-	-	-	-	-	√	-	-
TC.C-CP2	-	-	-	-	-	-	-	-	-	√	-
TC.C-CP3	-	-	-	-	-	-	-	-	√	-	-
TC.C-CP4	-	-	-	-	-	-	-	-	√	-	-
TC.C-UP1	-	-	-	-	-	√	-	√	-	-	-
TC.C-UP2	-	-	-	-	-	-	√	-	-	-	-
TC.C-UP3	-	-	-	-	-	-	-	√	-	-	-
TC.C-UP4	-	-	-	-	-	-	-	√	-	-	-
TC.C-UP5	-	-	-	-	-	-	-	√	-	-	-
TC.C-UP6	-	-	-	-	-	-	-	√	-	-	-
TC.C-UP7	-	-	-	-	-	-	-	-	-	-	√
TC.C-UP8	-	-	-	-	-	-	-	-	-	-	√

**Table 7:** Test result and vulnerability disclosure.

Index	Test Case	Operator A	Operator B	Operator C
TC. R1	RRC Connection DoS	O	O	X
TC. R2	RRC Security mode command	X	O	X
TC. R3	RRC Connection reconfiguration	X	O	X
TC.C-CP1	NAS Integrity Spoofing	O	O	O
TC.C-CP2	NAS Ciphering Spoofing	O	O	O
TC.C-CP3	NAS Security mode command	O	O	X
TC.C-CP4	NAS Attach accept	O	O	X
TC.C-UP1	EPC Scanning	X	O	X
TC.C-UP2	UE IP Depletion	X	X	X
TC.C-UP3	Targeted Create Session Request	X	X	X
TC.C-UP4	Delete Session Request	X	X	X
TC.C-UP5	SIP de-Register Request	X	X	X
TC.C-UP6	SIP Bye Request	X	X	X
TC.C-UP7	SIP Message Request	X	X	X
TC.C-UP8	MMS Request	X	X	X

**Table 8:** Vulnerability mitigation ^1^.

Test Case	Root Cause	Vulnerability Mitigation
RRC Connection DoS	Design Flaw	Security Guide Standardization
RRC Security mode command	Implementation Flaw	eNB Software PKG Patch
RRC Connection reconfiguration	Implementation Flaw	eNB Software PKG Patch
NAS Integrity Spoofing	Implementation Flaw	MME Configuration alteration
NAS Ciphering Spoofing	Design Flaw	Security Guide Standardization
NAS Security mode command	Implementation Flaw	MME Software PKG Patch
NAS Attach accept	Implementation Flaw	MME Software PKG Patch
EPC Scanning	Design Flaw	Intrusion Detection System

^1^ Patching the carrier’s equipment or standardization with ITU by discussing with 3 GPP are in progress for identified vulnerabilities.

## Data Availability

Not applicable.
